# Establishment and validation of two nomograms to predict the benefit of concurrent chemotherapy in stage II‐IVa nasopharyngeal carcinoma patients with different risk factors: Analysis based on a large cohort

**DOI:** 10.1002/cam4.2841

**Published:** 2020-01-10

**Authors:** Xue‐Song Sun, Bei‐Bei Xiao, Chao Lin, Sai‐Lan Liu, Qiu‐Yan Chen, Lin‐Quan Tang, Hai‐Qiang Mai

**Affiliations:** ^1^ Sun Yat‐sen University Cancer Center State Key Laboratory of Oncology in South China Collaborative Innovation Center for Cancer Medicine Guangdong Key Laboratory of Nasopharyngeal Carcinoma Diagnosis and Therapy Guangzhou P. R. China; ^2^ Department of Nasopharyngeal Carcinoma Sun Yat‐sen University Cancer Center Guangzhou P. R. China

**Keywords:** concurrent chemotherapy, nasopharyngeal carcinoma, nomogram, radiotherapy, survival

## Abstract

**Objective:**

We aimed to establish and validate two nomograms that predict progression‐free survival (PFS) and overall survival (OS) in patients with stage II–IVa nasopharyngeal carcinoma (NPC) while evaluating the benefit of concurrent chemotherapy.

**Patients and Methods:**

We randomly divided 3412 patients newly diagnosed with stage II‐IVa NPC between 2008 and 2013 into training and validation ‘A’ cohorts (n = 1706 each). Another set of patients diagnosed between 2014 and 2016 served as validation cohort ‘B’ (n = 1503). A Cox multivariate model using the backward stepwise approach was applied to develop the nomograms, which were assessed for accuracy (Harrel C index) and calibration.

**Results:**

The 3‐ and 5‐year PFS rates in the training cohort were 86.8% (95% confidence interval [CI] 85.0%‐88.6%) and 82.3% (95% CI 80.1%‐84.5%), respectively. For the PFS nomogram, 5 variables were selected based on a backward procedure in the multivariate Cox model (gender, T stage, N stage, Epstein‐Barr virus DNA, and treatment method). The same variables plus patient age and diabetes mellitus were used for the OS nomogram. The Harrell C indices of the training, validation A, and validation B cohorts were 0.711, 0.700, and 0.703, respectively, for PFS, and 0.775, 0.743, and 0.727, respectively, for OS. Both nomograms performed well in terms of calibration in the training and validation cohorts.

**Conclusions:**

Our nomograms are reliable prognostic predictors of PFS and OS in patients with stage II‐IVa NPC. These nomograms could robustly estimate an individual's benefit from concurrent chemotherapy, which assists in treatment decision‐making.

## Graphical abstract

Our nomograms provided reliable prognostic values in predicting PFS and OS in stage II‐IVa NPC patients. These nomograms could robustly estimate individual benefit from CCT, which strengthens decision‐making around prognosis.

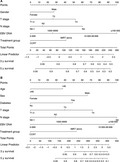

## 1 INTRODUCTION

Nasopharyngeal carcinoma (NPC) is a type of head and neck cancer with a high incidence rate of approximately 30 per 100 000 population in endemic areas such as southern Asia.^1^, ^2^ Owing to the high sensitivity of this cancer to irradiation, radiotherapy (RT) has become the primary treatment modality^3^; however, chemotherapy has been increasingly applied in recent years to improve these patients' survival rates. Based on data from previous clinical trials, concurrent chemoradiotherapy (CCRT) is now established as the standard treatment for locoregionally advanced NPC (LANPC).^4^

With advances in RT techniques, the overall survival (OS) of patients with NPC has considerably improved,^5^ and the role of concurrent chemotherapy (CCT) has therefore warranted reevaluation. The therapeutic value of CCT involving intensity‐modulated RT (IMRT) has remained unclear given that patient heterogeneity produced conflicting results.^6^, ^7^, ^8^, ^9^, ^10^ Therefore, it has become evident that risk assessment for individual clinical decision‐making is warranted if personalized, precision medicine is to be pursued; for example, a patient who is considered to have poor survival prospects before initial treatment may require a more aggressive therapeutic regimen.

Currently, risk assessment and treatment stratification for patients with NPC are primarily based on the tumor‐node‐metastasis (TNM) system. Despite this being the internationally recognized staging standard, it may not be clinically efficient since patients with the same TNM stage who undergo similar treatments have been shown to have varying clinical outcomes.^11^ Hence, more precise and comprehensive tools are required to identify individual risks by incorporating informative but currently underused prognostic factors that can then be developed to improve individual patient treatment.

Nomograms can be used to incorporate various risk factors to create a simple graphical model that predicts the prognoses of patients with cancer; they have been demonstrated to be useful tools in guiding treatments in clinical practice.^12^, ^13^ In this study, we aimed to construct nomograms that predict OS and progression‐free survival (PFS) in patients with stage II–IVa NPC who are at high risk of treatment failure. Moreover, we incorporated the treatment method (CCRT or RT alone) into these nomograms to intuitively estimate the benefit of CCT for individual patients.

## 2 MATERIALS AND METHODS

### 2.1 Patient population

**Figure 1:**
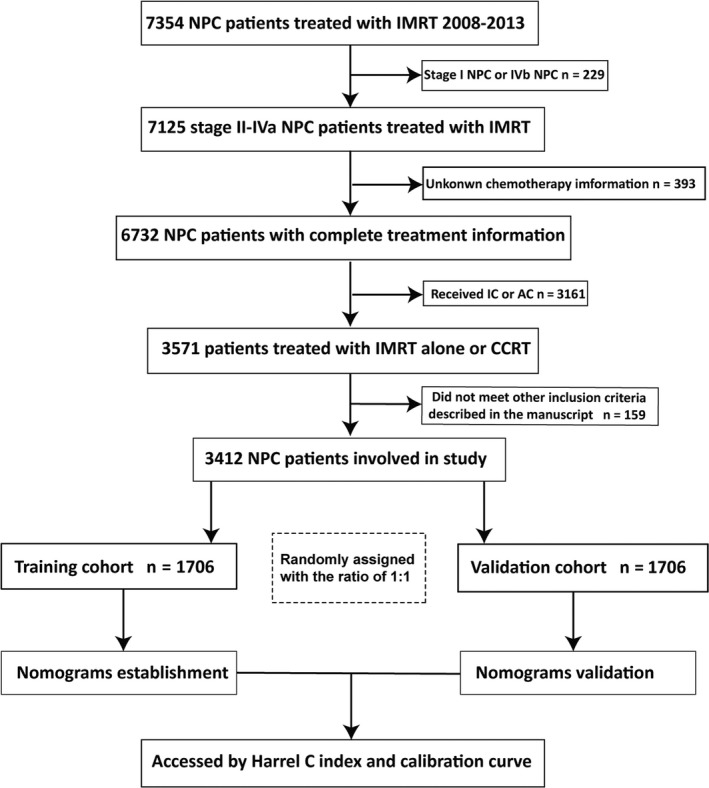
Flowchart showing the patient selection process. CCRT, concurrent chemoradiotherapy; IMRT, intensity‐modulated radiotherapy; NPC, nasopharyngeal carcinoma

A total of 4915 patients newly diagnosed with stage II‐IVa NPC between January 2008 and December 2016 were retrospectively investigated in this study based on the following inclusion criteria: (a) pathologically diagnosed NPC of World Health Organization type II or III; (b) clinical stage II–IVa based on the 8th American Joint Committee on Cancer (AJCC) staging system; (c) treated with IMRT with or without platinum‐based CCT; (d) available hematological sample results; (e) adequate hematologic hepatic and renal function; (f) no subsequent second malignant tumor or history of previous cancers; and (g) sufficient follow‐up data. All patients were restaged according to the 8th AJCC staging manual. Between January 2008 and December 2013, 3412 patients were randomly allocated in equal proportions into the training cohort (n = 1706) and validation cohort A (n = 1706) using computer software‐generated random numbers. A flowchart of the patient selection process is shown in Figure 1. A second cohort including patients diagnosed between January 2014 and December 2016 served as validation cohort B. The study was approved by the clinical research ethics committee of Sun Yat‐sen University Cancer Center (SYSUCC), Guangzhou, China, and all patients provided written informed consent before treatment.

### 2.2 Treatment and follow‐up

All eligible patients were treated based on the SYSUCC treatment protocol for NPC. Generally, all patients received IMRT at a total dose of 68‐70 Gy to the primary lesion and metastatic lymph node areas (2 Gy/day, 5 times per week). The regional lymphatic drainage area was irradiated with a total dose of 50‐54 Gy. For patients who were also treated with CCT during IMRT, cisplatin, carboplatin, or nedaplatin was administered simultaneously with RT using either a weekly or triweekly regimen.

After treatment, patients were required to attend follow‐up examinations every 3 months during the first 3 years and then every 6 months thereafter. Blood examinations (routine blood tests, blood biochemistry, and serum Epstein‐Barr virus [EBV] DNA levels), nasopharyngoscopy, head and neck magnetic resonance imaging, chest radiography, abdominal sonography, and bone scans were performed during follow‐up visits annually or when tumor relapse was suspected. Biopsies were performed to estimate suspicious relapse or metastatic lesions. The primary endpoint was PFS, which was defined as the interval between the first diagnosis and tumor relapse or detection of distant metastasis, death for any reason, or the date of the last follow‐up visit. The secondary endpoint was OS, which was calculated from the time between the date of first admission to that of death from any cause; otherwise, patients were censored from the date of the last follow‐up visit.

### 2.3 Statistical analysis

Survival rates were calculated using Kaplan‐Meier curves and compared using the log‐rank test. All characteristics were converted into categorical variables. Multivariate Cox proportional hazards models using the backward stepwise approach were used to identify potential prognostic factors. Variables with a 2‐tailed *P* < .05 were considered statistically significant and were subsequently incorporated into the nomogram model. Finally, nomograms that predicted 3‐ and 5‐year PFS and OS were developed; the concordance index (C‐index) was used to assess their accuracy and was calculated using the bootstrap‐corrected method based on 1000 resamples. Calibration curves that compared the observed Kaplan‐Meier data with the survival probabilities predicted by the nomograms were formulated to evaluate the reliabilities of the nomograms. All analyses were performed using SPSS version 23.0 (IBM Corp.) or R version 3.5.0 (R Core Team, Vienna, Austria).

## 3 RESULTS

### 3.1 Patient characteristics and survival

The median patient age in the training cohort was 46 years (range, 18‐59 years) with a male‐to‐female ratio of 2.7:1. Overall, 6.7%, 28.0%, 52.3%, and 13.0% of the patients had stage T1, T2, T3, and T4, respectively, whereas 22.7%, 43.8%, 29.2%, and 4.2% had stage N0, N1, N2, and N3, respectively. The characteristics of the patients in each cohort are listed in Table 1.

**Table 1:** Patient characteristics

Characteristic	Training cohort	Validation cohort A	Validation cohort B
Total	1706	1706	1503
Age (y)			
≤46	824 (48.3%)	822 (48.2%)	768 (51.1%)
>46	882 (51.7%)	884 (51.8%)	735 (48.9%)
Gender			
Female	467 (27.4%)	480 (28.1%)	467 (31.1%)
Male	1239 (72.6%)	1226 (71.9%)	1036 (68.9%)
Smoking history			
No	1051 (61.6%)	1043 (61.6%)	1075 (71.5%)
Yes	655 (38.4%)	663 (38.9%)	428 (28.5%)
Nasopharyngeal carcinoma family history			
No	1500 (87.9%)	1501 (88.0%)	1328 (88.4%)
Yes	206 (12.1%)	205 (12.0%)	175 (11.6%)
Diabetes mellitus			
No	1652 (96.8%)	1659 (97.2%)	1452 (96.9%)
Yes	54 (3.2%)	47 (2.8%)	51 (3.4%)
Cardiovascular disease			
No	1595 (93.5%)	1591 (93.3%)	1360 (90.5%)
Yes	111 (6.5%)	115 (6.7%)	143 (9.5%)
T stage^a^			
T1	115 (6.7%)	132 (7.7%)	140 (9.3%)
T2	477 (28.0%)	450 (26.4%)	281 (18.7%)
T3	892 (52.3%)	865 (50.7%)	882 (58.7%)
T4	222 (13.0%)	259 (15.2%)	200 (13.3%)
N stage^a^			
N0	387 (22.7%)	363 (21.3%)	265 (17.6%)
N1	748 (43.8%)	747 (43.8%)	636 (42.3%)
N2	499 (29.2%)	535 (31.4%)	474 (31.5%)
N3	72 (4.2%)	61 (3.6%)	128 (8.5%)
Epstein‐Barr virus DNA level (copies/mL)			
<1000	863 (50.6%)	825 (48.4%)	801 (53.3%)
1000‐9999 copies/ml	402 (23.6%)	421 (24.7%)	381 (25.3%)
10 000‐99 999 copies/ml	281 (16.5%)	284 (16.6%)	198 (13.2%)
≥100 000	160 (9.4%)	176 (10.3%)	123 (8.2%)
Treatment group			
IMRT alone	532 (31.2%)	493 (28.9%)	330 (22.0%)
CCRT	1174 (68.8%)	1213 (71.1%)	1173 (78.0%)

Abbreviations: CCRT, concurrent chemoradiotherapy; IMRT, intensity‐modulated radiotherapy.

a According to the 8th edition of the Union of International Cancer Control/American Joint Committee on Cancer staging system.

The median follow‐up for the training cohort was 47.8 months (interquartile range [IQR] 33.3‐66.2 months); the 3‐ and 5‐year PFS rates were 86.8% (95% confidence interval [CI] 85.0%‐88.6%) and 82.3% (95% CI 80.1%‐84.5%), respectively. In validation cohort A, the median follow‐up was 46.7 months (IQR 32.3‐64.6 months); the 3‐ and 5‐year PFS rates were 86.4% (95% CI 84.6%‐88.2%) and 82.4% (95% CI 80.2%‐84.6%), respectively. The median follow‐up for validation cohort B was 40.8 months (IQR 28.0‐59.1 months); the 3‐ and 5‐year PFS rates were 88.3% (95% CI 87.1%‐89.4%) and 84.1% (95% CI 82.3%‐85.9%), respectively.

### 3.2 Establishment and validation of a nomogram model for PFS

**Figure 2:**
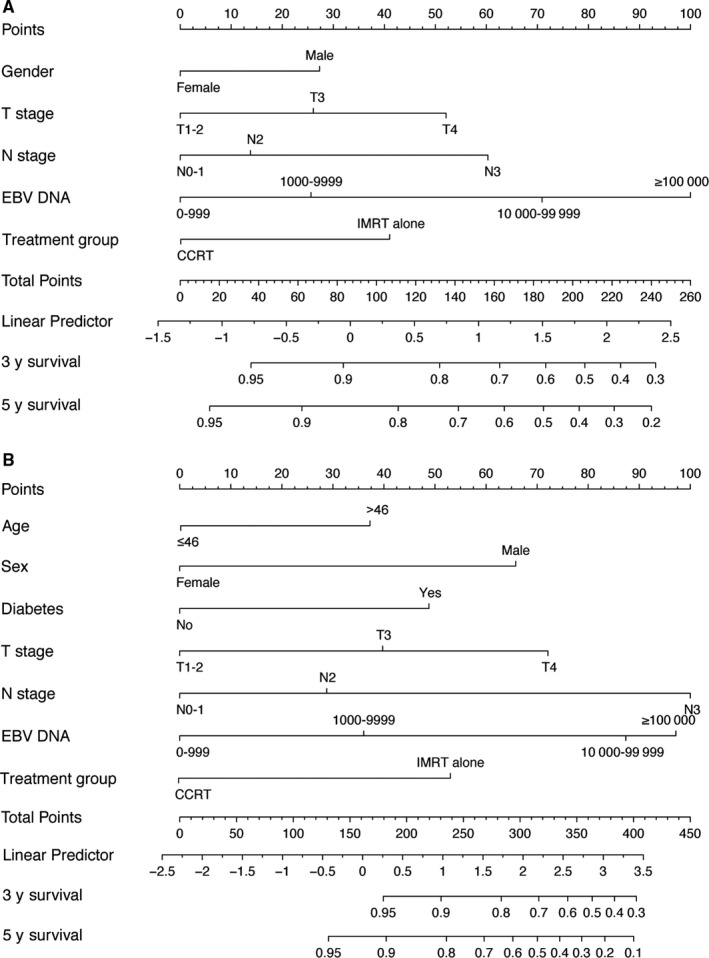
A, Nomogram for predicting 3‐ and 5‐y progression‐free survival (PFS) in patients with stage II‐IVa nasopharyngeal carcinoma (NPC). As an example, locate the patient's gender and draw a line straight up to the “Points” axis to determine the score associated with that gender. Add the scores achieved for each covariate, and locate this sum on the “Total Points” axis. Draw a line straight down to determine the likelihoods of 3‐ and 5‐y PFS. B, Nomogram for predicting 3‐ and 5‐y overall survival (OS) in patients with stage II–IVa NPC. As an example, locate the patient's gender and draw a line straight up to the “Points” axis to determine the associated score. Add the scores achieved for each covariate, and locate this sum on the “Total Points” axis. Draw a line straight down to determine the likelihood of 3‐ or 5‐y OS. CCRT, concurrent chemoradiotherapy; EBV, Epstein‐Barr virus; IMRT, intensity‐modulated radiotherapy

**Figure 3:**
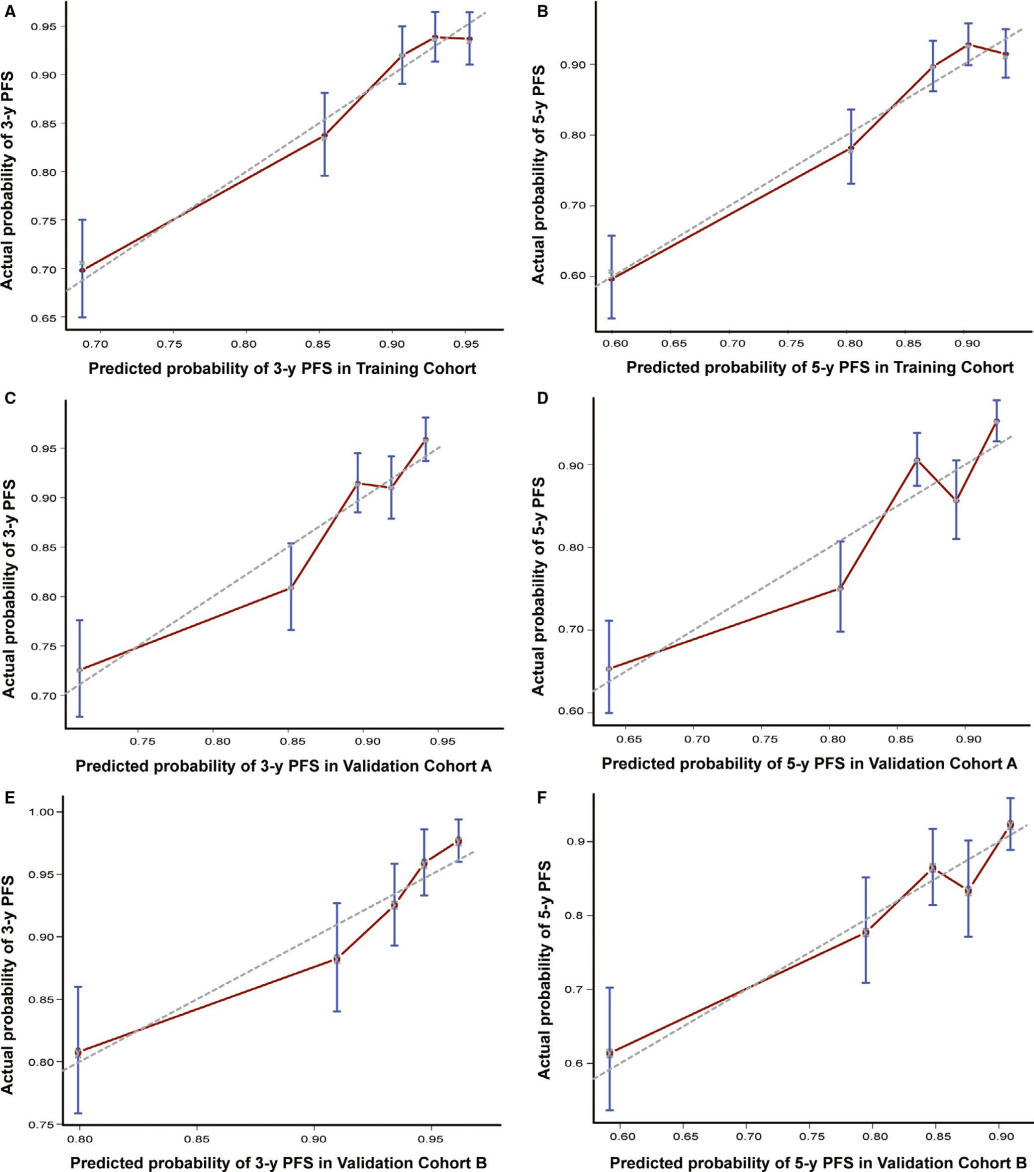
The calibration curves for predicting patient progression‐free survival (PFS). (A) Three‐year PFS in training cohort, (B) 5‐y PFS in training cohort, (C) 3‐y PFS in validation cohort A, (D) 5‐y PFS in validation cohort A, (E) 3‐y PFS in validation cohort B, and (F) 5‐y PFS in validation cohort B

In the training cohort, all potential prognostic factors (age, gender, smoking, family history of NPC, T stage, N stage, diabetes mellitus, cardiovascular disease, EBV DNA, and treatment method) were considered in the multivariate Cox model using the backward stepwise approach for the selection of variables. Five of these factors (gender, T stage, N stage, EBV DNA, and treatment method) significantly influenced PFS (*P* < .05; Table 2) and were used to establish a nomogram for predicting 3‐ and 5‐year PFS (Figure 2A). Each variable represented a score corresponding to the point scale, and total scores were calculated by summing the scores of each variable. Next, PFS probabilities were estimated at the 3‐ and 5‐year time points by projecting the total scores on a probability scale. Using the bootstrap validation method, the Harrell C index for the nomogram was 0.711 (95% CI 0.678‐0.744) in the training cohort, which exhibited satisfactory accuracy for predicting 3‐ and 5‐year PFS. Furthermore, the Harrell C indices were 0.700 (95% CI 0.670‐0.730) in validation cohort A and 0.703 (95% CI 0.664‐0.742) in validation cohort B. The calibration curves indicated that the nomogram showed acceptable agreement between the nomogram‐predicted and actual values for 3‐ and 5‐year PFS in the 3 cohorts (Figure 3).

**Table 2:** Multivariate analysis of prognostic factors for PFS and OS

Characteristic	HR	95% CI	*P* value
PFS			
Age	1.256	0.975‐1.618	.077
Gender	1.511	1.110‐2.056	.009
Family history of NPC	0.688	0.444‐1.067	.095
Diabetes mellitus	1.598	0.925‐2.761	.093
T stage			
T3 vs T1‐2	1.445	1.071‐1.950	.016
T4 vs T1‐2	2.101	1.449‐3.048	<.001
N stage			
N2 vs N0‐1	1.232	0.932‐1.629	.143
N3 vs N0‐1	2.452	1.535‐3.918	<.001
EBV‐DNA level			
1000‐9999 vs < 1000	1.496	1.038‐2.156	.031
10 000‐99 999 vs < 1000	2.960	2.094‐4.185	<.001
≥100 000 vs < 1000	4.480	3.128‐6.416	<.001
Treatment method	0.556	0.422‐0.732	<.001
Overall survival			
Age	1.689	1.197‐2.384	.003
Gender	2.536	1.594‐4.037	<.001
Diabetes mellitus	1.994	1.088‐3.657	.026
T stage			
T3 vs T1‐2	1.754	1.174‐2.620	.006
T4 vs T1‐2	2.774	1.729‐4.451	<.001
N stage			
N2 vs N0‐1	1.503	1.050‐2.150	.026
N3 vs N0‐1	4.113	2.368‐7.144	<.001
EBV‐DNA level			
1000‐9999 vs < 1000	1.664	1.013‐2.735	.044
10 000‐99 999 vs < 1000	3.441	2.176‐5.441	<.001
≥100 000 vs < 1000	3.952	2.428‐6.433	<.001
Treatment method	0.471	0.333‐0.667	<.001

Note

A Cox proportional hazards model was used to conduct multivariate analyses. All variables were transformed into categorical variables. HRs were calculated for age (>46 y vs ≤46 y); gender (male vs female); smoking (yes vs no); family history of NPC (yes vs no); diabetes mellitus (yes vs no); cardiovascular disease (yes vs no); and treatment method (concurrent chemoradiotherapy vs intensity‐modulated radiotherapy alone).

We selected variables using the backward stepwise approach. The *P* value threshold was .1 (*P* > .1) for the removal of insignificant variables from the model. Only variables significantly associated with survival were included in the further analysis.

Abbreviations: CI, confidence interval; EBV, Epstein‐Barr virus; HR, hazard ratio; NPC, nasopharyngeal carcinoma; PFS, progression‐free survival.

### 3.3 Establishment and validation of a nomogram model for OS

**Figure 4:**
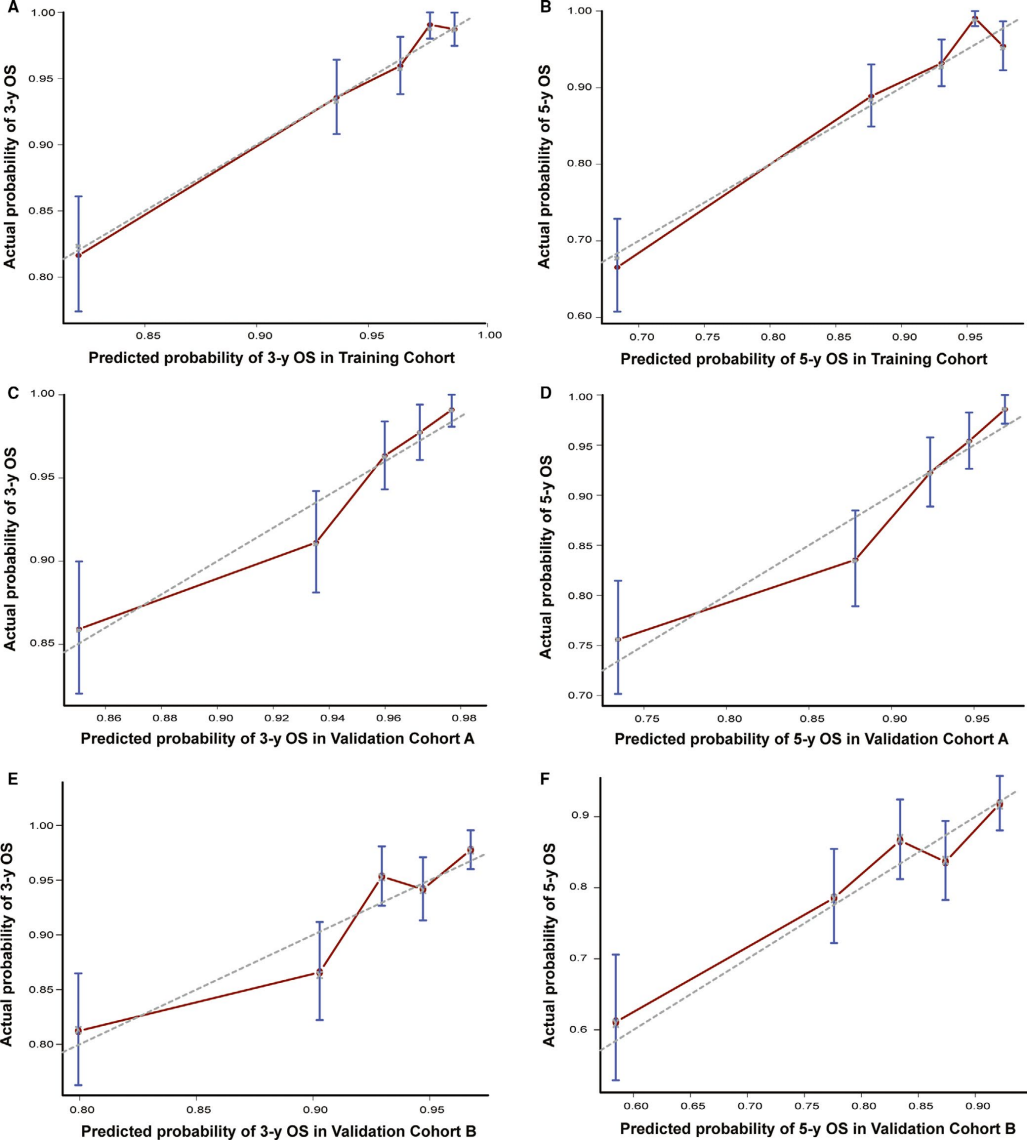
The calibration curves for predicting patient overall survival (OS). (A) Three‐year OS in training cohort, (B) 5‐y OS in training cohort, (C) 3‐y OS in validation cohort A, (D) 5‐y OS in validation cohort A, (E) 3‐y OS in validation cohort B, and (F) 5‐y OS in validation cohort B

We further constructed and validated the OS nomogram model using the same method. As shown in Table 2, all backward selected variables (age, gender, diabetes mellitus, T stage, N stage, EBV DNA, and treatment method) significantly influenced OS and were included in the nomogram model (Figure 2B). The Harrell C indices for the OS nomogram were 0.775 (95% CI 0.738‐0.812) in the training cohort, 0.743 (95% CI 0.706‐0.780) in validation cohort A, and 0.727 (95% CI 0.668‐0.786) in validation cohort B. Calibration curves for 3‐ and 5‐year OS revealed good correlations between the nomogram's estimated values and the actual value (Figure 4).

## 4 DISCUSSION

We established PFS and OS nomograms for patients with stage II‐IVa NPC who received radical IMRT. Patients with stage I NPC were excluded from the study owing to their low tumor burdens and their having been treated with IMRT alone based on the NCCN guidelines.^14^ By using these two nomograms, the prognosis of individual patients with different risk factors could easily be predicted; moreover, they may be particularly useful for estimating the benefit of CCT on an individual basis, which may be pivotal for guiding individual treatment. Both nomograms were successfully validated using cohorts from the same period as that of the training cohort as well as a later period.

The Intergroup‐0099 (INT‐0099) trial was the first to demonstrate that the addition of chemotherapy to RT could significantly improve both OS and PFS in patients with LANPC compared with RT alone.^15^ In an Asian cohort, Lin et al directly compared CCRT with RT alone and demonstrated the curative effect of the former.^4^ Therefore, CCRT has been established as the standard treatment for such patients. Our group conducted the only randomized controlled trial for patients with stage II NPC to date, in whom we also demonstrated that the addition of CCT to RT could further improve survival outcomes.^16^ Notably, all the aforementioned studies were performed in the 2‐dimensional RT era. As IMRT achieves a more accurate dose distribution and further prolongs patient survival, the role of CCT needs to be reevaluated.^17^, ^18^, ^19^

In the IMRT era, there have been several studies comparing the survival outcomes of patients with NPC who received RT alone to those who underwent CCRT.^6^, ^9^, ^10^, ^20^, ^21^, ^22^ Owing to the diversity of conditions and comorbidities of patients included in these studies, the conclusions regarding the role of CCT varied. According to a meta‐analysis of patients with stage II NPC, those receiving IMRT alone achieved equivalent survival outcomes to those who underwent CCRT, with fewer grade 3‐4 acute toxicities.^9^ Cao et al reviewed 117 patients with LANPC (T4 classification) and concluded that IMRT alone achieved similar OS outcomes to CCRT,^22^ whereas Sun et al found that CCRT was superior to IMRT alone for patients with advanced N stage (N2 and N3) NPC.^10^ Liang et al evaluated the benefits of CCT against NPC with different EBV‐DNA levels. Interestingly, only patients with higher EBV‐DNA levels (>4000 copies/mL) benefitted from additional CCT.^6^ These studies corroborated the fact that the role of CCT differs among patients with dissimilar tumor burdens. Because of the toxicity of CCT, a high incidence of grades III‐IV adverse events was observed^23^; therefore, risk assessment for each patient before treatment is necessary for guiding individualized treatment.

In this respect, the current AJCC/Union of International Cancer Control stage classification for NPC has been found to be of limited value to clinicians because of several limitations. Many prognostic aspects verified in previous studies are ignored by this system; these include age, gender, comorbidities, and particularly EBV DNA, which is an important biomarker for NPC diagnosis.^24^, ^25^, ^26^ Therefore, we established two nomograms that incorporated these potential prognostic factors to predict the prognoses of these patients more accurately. Moreover, the treatment methods (IMRT alone or CCRT) were incorporated into the model, which enables us to assess the benefit of CCT individually in patients with different risk factors.

The strength of our nomograms lay in the large cohort upon which they were based. Moreover, all patients received IMRT, which is the contemporary RT method used worldwide. Our two nomograms showed satisfactory discrimination and good calibration in the training and validation cohorts. Using the backward stepwise procedure, 5 factors were taken into consideration for the PFS nomogram. Consider the following examples that illustrate the value of this model in terms of individualized treatment: A woman (0 points) with T3 (27 points), N0 stage (0 points) NPC, and with an EBV‐DNA level of 500 copies/mL (0 point) but no diabetes would obtain a total score of 27 or 71 depending on whether she received CCRT or IMRT alone, yielding an estimated 3‐year PFS rate of 95% or 92%, respectively; as such, the corresponding benefit of CCT is 3%. Considering the potential treatment‐related toxicity, cost, and inconvenience of chemotherapy, IMRT alone would be the most appropriate option for this patient. However, a man (28 points) with T4 (52 points), N3 (60 points), and an EBV‐DNA level of 5000 copies/mL (60 points) who receives CCRT (0 points) would have a total score of 172 and a corresponding 3‐year PFS of 75%. If the patient receives IMRT alone, the total score would be 213 points and the estimated 3‐year PFS would fall to 50%. In this case, it is clearly more beneficial (by a 25 percentage point advantage) for this patient to receive CCRT. The OS benefits obtained from CCT can also be calculated in the same manner. In the nomogram for OS, older age and diabetes were also associated with a worsened prognosis, which may be attributable to OS being more easily influenced by age and unrelated concurrent diseases, as these are not related to the clinical course of NPC progression per se.

Although the nomograms demonstrated good accuracy for predicting PFS and OS, there were several limitations to this study. First, this was a retrospective investigation with an unavoidable selective bias. Although not statistically significant, there are certainly important clinicopathologic differences between the patients who received CCT and those who did not. Besides, as some information was not available for all patients, the adverse effects of CCT (which would almost certainly vary among patients) were not considered in our study. Second, all patients were from a single treatment center in an endemic area, and our models were not validated in an external cohort. Third, there is no standardized global consensus for the measurement of plasma EBV DNA. Finally, the C‐indices of the nomograms were only 0.700‐0.743 in the validation cohorts; therefore, more factors should be considered in future studies to develop a more complete model.

## CONFLICT OF INTERESTS

The authors declare that they have no conflict of interest.

## AUTHOR CONTRIBUTIONS

Study concepts: Hai‐Qiang Mai, Lin‐Quan Tang, Qiu‐Yan Chen; study design: Xue‐Song Sun, Bei‐Bei Xiao, Chao Lin, Sai‐Lan Liu; data acquisition: Xue‐Song Sun, Bei‐Bei Xiao, Chao Lin, Sai‐Lan Liu; quality control of data and algorithms: Xue‐Song Sun, Bei‐Bei Xiao, Chao Lin; data analysis and interpretation: Xue‐Song Sun, Bei‐Bei Xiao, Chao Lin, Sai‐Lan Liu; statistical analysis: Xue‐Song Sun, Bei‐Bei Xiao, Chao Lin; manuscript preparation: Xue‐Song Sun, Bei‐Bei Xiao, Chao Lin; manuscript editing: Xue‐Song Sun, Bei‐Bei Xiao, Chao Lin; manuscript review: Hai‐Qiang Mai, Lin‐Quan Tang, Qiu‐Yan Chen.

## Data Availability

The datasets used and/or analyzed during the current study are available from the corresponding author on reasonable request.
